# Reflections on speech-language-hearing therapy in obesity and bariatric surgery

**DOI:** 10.1590/2317-1782/e20240364en

**Published:** 2025-10-13

**Authors:** Sarah Letycia de Sá Crespo Albuquerque Costa, Ithalo José Alves da Silva Cruz, Pedro Manoel Araújo de Santana, Maria das Graças Duarte, Kelli Nogueira Ferraz Pereira Althoff

**Affiliations:** 1 Programa de Pós-Graduação em Saúde da Comunicação Humana, Universidade Federal de Pernambuco – UFPE - Recife (PE), Brasil.; 2 Hospital das Clínicas, Universidade Federal de Pernambuco – UFPE - Recife (PE), Brasil.

Dear editors,

I would like to submit for consideration by the CoDAS Editorial Board a scientific reflection on the growing relevance of speech-language-hearing (SLH) therapy in managing obesity and monitoring patients undergoing bariatric surgery (BS).

Obesity is a chronic disease characterized by fat accumulation, generating an inflammatory state that culminates in increased morbidity and mortality^(1,2)^. Its global prevalence has increased substantially over the last 40 years, from less than 1% in 1975 to 6-8% in 2016, and is expected to rise to 33% by 2030^(2,3)^. Estimates for severe obesity raise even greater concern, as it is expected to increase by 130% worldwide. Statistics also indicate that 57.2% of the Brazilian population is overweight and that 54% of adults will have a high BMI by 2035^(4)^. Thus, more than half of the global population considered healthy will be overweight by 2035, requiring effective public health policies and properly trained professionals to understand and manage obesity.

Its treatment includes approaches such as exercise, anti-obesity medications, and nutritional monitoring. However, many individuals who fail conventional treatments undergo BS, which has proven to be the most effective procedure for treating severe obesity^(5,6)^.

Obese individuals referred for BS experience several changes in stomatognathic structures and functions. These include damage to the lips, teeth, muscles, pharynx, and larynx, impairing breathing, phonation, mastication, and swallowing. Excessive accumulation of adipose tissue in the oral cavity, pharynx, and larynx exerts pressure on these structures, affecting their mobility and functional capacity. This fat accumulation can reduce muscle efficiency, hindering sound articulation, mastication, and airflow, impacting breathing and swallowing, thus making these functions less effective. Studies have linked overweight individuals to a specific mastication style, characterized by larger bite sizes and faster eating, compared to normal-weight adults^(7-9)^.

Despite these recommendations, BS is often proposed for obese patients, regardless of their dental condition and mastication ability. Considering the crucial preoperative preparation, BS candidates must undergo masticatory reeducation, as such training can help obese patients lose weight and improve metabolic function^(10)^.

SLH pathologists are the professionals responsible for human communication health regarding the promotion, prevention, and recovery of orofacial functions, voice, speech, and language. They may work in cooperation with other professionals^(10)^. Hence, the role of SLH pathologists is increasingly essential and of paramount importance in multidisciplinary BS teams.

Due to its relevance, the technical opinion on the Role of SLH Pathologists in the Clinical Treatment of Obesity and BS^(11)^ considers their work essential in the clinical treatment of obesity and BS concerning the assessment and diagnosis of oral-motor disorders. Evidence of the changes that will occur in the lives of patients after bariatric surgery, particularly regarding nutrition, highlights the need for speech-language pathologists to provide support from the pre-surgical stage, aiming to improve the quality of life of these individuals, who will need to adapt to the new way of eating, with emphasis on masticatory mechanics.

The Brazilian Society of Bariatric and Metabolic Surgery^(12)^ recommends that the postoperative diet go through stages from liquid, pureed, and soft to regular food. Masticatory function is crucial during these phases, as food intolerance, characterized by frequent postprandial vomiting, constipation, and hiccups, is the main consequence of a lack of presurgical guidance to such patients.

Thus, SLH intervention in patients undergoing BS is beneficial mainly regarding the rehabilitation of stomatognathic functions and the improvement of post-surgery quality of life as the patient adapts to BS, adjusting oral myofunctional and phonatory structures and functions^(13)^. The change in ingested volume and gastric emptying rate after surgery requires learning the new eating pattern^(14)^. SLH monitoring is essential for the safe and effective return of food consistencies and textures, avoiding complications such as choking, vomiting, and food stasis, favoring bariatric success and improved quality of life^(15)^.

Even though SLH pathologists’ participation in multidisciplinary teams monitoring BS patients is fairly recent, the literature has approached their role in these patients for over a decade^(16)^. Nevertheless, the inclusion of SLH pathologists in multidisciplinary teams monitoring obesity is still incipient in many regions of Brazil. In 2020, Tomanchieviez et al.^(17)^ proposed a study to determine patients' perceptions of BS preoperative and postoperative SLH care. Their results showed that 35.48% of the participants were unaware of the role of SLH pathologists in BS and only understood their importance after receiving guidance and intervention; these same participants rated their role as “extremely significant” or “significant.”

Their work is even more relevant considering the Good Health and Well-being goal among the UN’s 17 Sustainable Development Goals (SDGs). It focuses on food and the interconnected aspects of the economy, society, environment, and, above all, the factors involved in diseases such as obesity and associated comorbidities^(18)^.

Lastly, we reiterate the importance of having the scientific community discuss widely the role of SLH pathologists in such a challenging and impactful public health scenario. I hope this reflection will encourage debate and further research in this field.
