# Supplementary material for: Reflections on speech-language-hearing therapy in obesity and bariatric surgery
Source: Codas. 2025 Oct 13;37(5):e20240364. doi: 10.1590/2317-1782/e20240364en (PMC12533525; doi:10.1590/2317-1782/e20240364en)
Supplement: Translation

# Translation: Reflexão sobre a atuação fonoaudiológica na obesidade e cirurgia bariátrica

**DOI:** 10.1590/2317-1782/e20240364pt

**Authors:** Sarah Letycia de Sá Crespo Albuquerque Costa, Ithalo José Alves da Silva Cruz, Pedro Manoel Araújo de Santana, Maria das Graças Duarte, Kelli Nogueira Ferraz Pereira Althoff

**Affiliations:** 1 Programa de Pós-Graduação em Saúde da Comunicação Humana, Universidade Federal de Pernambuco – UFPE - Recife (PE), Brasil.; 2 Hospital das Clínicas, Universidade Federal de Pernambuco – UFPE - Recife (PE), Brasil.

Prezados Editores,

Gostaria de submeter para apreciação do Conselho Editorial da CODAS uma reflexão científica sobre a crescente relevância da atuação fonoaudiológica no manejo da obesidade e no acompanhamento de pacientes submetidos à cirurgia bariátrica.

A obesidade é uma doença crônica caracterizada pelo acúmulo de gordura, gerando um estado inflamatório que culmina em aumento da morbidade e mortalidade^(1,2)^. Sua prevalência global aumentou substancialmente nos últimos 40 anos, de menos de 1% em 1975, para 6-8% em 2016, e deve aumentar para 33% até 2030^(2,3)^. Para obesidade grave, as estimativas são mais preocupantes, com aumento esperado de 130% em todo o mundo. No Brasil, as estatísticas indicam que 57,2% da população está acima do peso e, até 2035 1,9% dos adultos terão IMC elevado^(4)^. Assim, até 2035, mais da metade da população considerada saudável mundial estará acima do peso, e políticas de saúde pública efetivas devem ser colocadas em prática, bem como os profissionais devem ser devidamente capacitados para compreender e manejar a obesidade.

Seu tratamento abrange abordagens como a prática de exercícios físicos, medicamentos antiobesidade e acompanhamento nutricional. Todavia, muitos indivíduos, por não responderem aos tratamentos convencionais, passam pela cirurgia bariátrica (CB), que tem se mostrado o procedimento mais eficaz para o tratamento da obesidade grave^(5,6)^.

Indivíduos obesos indicados para cirurgia bariátrica apresentam diversas alterações nas estruturas e funções estomatognáticas. Essas modificações incluem comprometimento dos lábios, dentes, músculos, faringe e laringe, com consequentes prejuízos nas funções de respiração, fonação, mastigação e deglutição. O acúmulo excessivo de tecido adiposo na cavidade oral, bem como nas regiões faríngea e laríngea, exerce pressão sobre essas estruturas, alterando sua mobilidade e capacidade de funcionamento. Esse acúmulo de gordura pode reduzir a eficiência muscular, dificultando a articulação dos sons e a mastigação, além de prejudicar a passagem do ar, impactando a respiração e a deglutição, tornando essas funções menos eficazes. Quanto à mastigação, estudos têm relacionado um estilo específico de mastigação para os indivíduos com excesso de peso, caracterizado por maior tamanho de mordida e uma alimentação mais rápida em comparação a adultos eutróficos^(7-9)^.

Apesar dessas recomendações, a CB é frequentemente proposta para pacientes obesos, independentemente da sua condição dentária e capacidade de mastigação. Considerando que o preparo pré-operatório do candidato à CB é de grande importância, é necessário que ele se submeta a uma reeducação mastigatória, visto que o treino com a função mastigatória para pacientes obesos pode ajudá-los na perda de peso e melhorar a função metabólica^(10)^.

O fonoaudiólogo é o profissional responsável pela saúde da comunicação humana, no que diz respeito à promoção, prevenção e recuperação das funções orofaciais, voz, fala e linguagem, podendo atuar de forma integrada com outros profissionais^(10)^. Na equipe multidisciplinar da cirurgia bariátrica, o papel do fonoaudiólogo vem se tornando cada dia mais fundamental e de suma importância.

Devida a relevância, o parecer técnico sobre a Atuação do Fonoaudiólogo no Tratamento Clínico da Obesidade e da Cirurgia Bariátrica^(11)^ considera necessária atuação do fonoaudiólogo no tratamento clínico da obesidade e da cirurgia bariátrica, no que se refere à avaliação e ao diagnóstico dos distúrbios da motricidade orofacial. As evidências das mudanças que ocorrerão na vida dos pacientes pós-cirurgia bariátrica, principalmente quanto à alimentação, levam à necessidade da contribuição fonoaudiológica com atuação desde o pré-cirúrgico, objetivando uma melhor qualidade de vida desses indivíduos, que necessitarão de adaptação à nova maneira de ingestão dos alimentos, dando destaque para a mecânica mastigatória.

A Sociedade Brasileira de Cirurgia Bariátrica e Metabólica^(12)^ preconiza que a dieta pós-operatória deve passar por estágios que vão desde a consistência líquida, pastosa, branda, até chegar na alimentação regular. Nessas fases, a função mastigatória exerce grande importância, uma vez que as principais consequências da falta de orientação pré-cirurgia nesses pacientes são a intolerância alimentar, caracterizada com vômitos, entalos ou soluços frequentes no período pós prandial.

Isto é, a atuação fonoaudiológica em pacientes submetidos à CB é benéfica principalmente no que diz respeito à reabilitação de funções estomatognáticas e à melhoria da qualidade de vida pós-cirurgia, quanto ao processo de adaptação à CB, adequando as estruturas e funções oromiofuncionais e fonatórias^(13)^, visto que a alteração do volume ingerido e a velocidade de esvaziamento gástrico após a cirurgia fazem com que seja necessário um aprendizado para o novo modo de alimentação^(14)^. O acompanhamento fonoaudiológico é essencial para que a adequação e o retorno das consistências e texturas alimentares sejam alcançados com segurança e eficácia, evitando complicações como engasgos, vômitos e estase do alimento, favorecendo o sucesso bariátrico e a melhoria da qualidade de vida^(15)^.

Embora a participação do fonoaudiólogo na equipe multidisciplinar do acompanhamento de pacientes candidatos a CB ainda seja recente, há literatura que mostra evidências da sua função nesses pacientes há mais de uma década^(16)^ Porém, destaca-se que a inserção do fonoaudiólogo em equipes multidisciplinares que acompanham a obesidade ainda é incipiente em muitas regiões do Brasil. Em 2020, Tomanchieviez e colaboradores^(17)^ propuseram um estudo com o objetivo de saber a percepção que os pacientes têm do atendimento do fonoaudiólogo no pré e pós-operatório da cirurgia bariátrica. Seus resultados mostraram que 35,48% dos sujeitos desconheciam a atuação do fonoaudiólogo na CB e só compreenderam a sua importância após receberem orientação e intervenção; os mesmos sujeitos avaliaram a atuação como “extremamente significativa” ou “significativa”.

A relevância dessa atuação ganha ainda mais força quando consideramos a meta de Saúde e Bem-estar dentre os 17 objetivos de desenvolvimento sustentável (ODS) desenvolvidos pela ONU, com foco na alimentação e nos aspectos interligados da economia, sociedade e meio ambiente; e, sobretudo, os fatores envolvidos em doenças como a obesidade e comorbidades associadas^(18)^.

Por fim, reiteramos a importância de discutir amplamente na comunidade científica o papel do fonoaudiólogo em um cenário tão desafiador e impactante para a saúde pública. Espero que esta reflexão contribua para fomentar debates e incentivar novas pesquisas na área.
